# Role of corporate social responsibility, management practices, organizational behavior in social equity with mediating role of women empowerment: A study of the Islamic banking sector of South Punjab

**DOI:** 10.3389/fpsyg.2022.926169

**Published:** 2022-10-13

**Authors:** Muhammad Farhan Basheer, Waseem Ul Hameed, Majid Ibrahim Mohammed Abdullah Al Zarooni, Rabeeya Raoof, Javeria Sattar

**Affiliations:** ^1^Lahore Business School, The University of Lahore, Lahore, Pakistan; ^2^Centre of Excellence for Islamic Finance and Social Equity (CEIFSE), Department of Islamic and Conventional Banking (DICB), Institute of Business Management and Administrative Sciences (IBMAS), The Islamia University of Bahawalpur (IUB), Bahawalpur, Pakistan; ^3^School of International Studies (SOIS), University Utara, Changlun, Malaysia

**Keywords:** corporate social responsibility, management practices, organizational behavior, women empowerment, social equity

## Abstract

The objective of this study was to identify the relationship between corporate social responsibility (CSR), management practices (MPs), and organizational behavior (OB) in social equity (SE) with the mediating role of women empowerment in the Islamic banking sector of South Punjab. It is a fact that, in the Islamic banking sector, South Punjab is facing a crisis in terms of SE for its employees and customers. This study is based on the quantitative data collected with a five-point Likert scale to identify the relationship between the variables of the framework. Smart PLS 3 software was used in this study to measure the primary data collected by 355 questionnaires. The study concluded that there is an important role of CSR, MPs, and OB in the management of SE in the Islamic banking sector of South Punjab. The theoretical framework of this study is a significant contribution to knowledge as it is based on the significant variables for SE. The study is significant because it provides theoretical as well as practical implications quite useful for the Islamic banking sector of South Punjab to cope with the problem of SE.

## Introduction

In the current era, it is observed that social values are decreasing in different organizations, and due to this problem, social equity (SE) is at stake. In this regard, the role of bad management and less attention to organizational values is noted as the key factors that are affecting the foundation of the organizations. The performance of the organizations has declined not only in the other business sector but also in the banking sector, which has challenged the norms and values of SE (Wut et al., 2022). Particularly, the Islamic banking sector of South Punjab is facing these problems, and several studies explained that ill management practices (MPs) are one of the core problems behind such kinds of issues. However, different studies have already been conducted in this regard, but these studies paid less attention to the issue of SE in the context of Islamic banking in South Punjab (García-Sánchez et al., 2022).

Corporate social responsibility (CSR) is when organizations are working in the light of sustainable goals triggered by the guidelines of sustainable development. It is the responsibility of every organization to work effectively to provide the maximum benefit to the community to ensure that the people of the community are not facing any kind of bias related to SE (Mathende and Nhapi, 2017). In the same way, MPs are the working pattern of the people of the management in any organization to ensure that everything is going according to the vision and mission of the organization. However, the MPs are different in each situation, and the bad MPs are creating problems for SE in a destructive way (Dathe et al., 2022). Similarly, according to Ahmad et al. (2022), organizational behavior (OB) refers to the working actions of the employees of any organization as a whole, in which the working environment and the performance of the employees are noted along with their attitude and individual performance. The world-class organizations have OB as a competitive advantage to satisfy the customers with a positive attitude and response. In addition, women empowerment (WE) refers to how women in any society are provided with the opportunity to obtain all the facilities of their life (Wijaya et al., 2022). In this way, it is important to provide equal opportunities to the women of the society for improving their standard of living. In addition, SE is when the individuals of the society are getting all the rights in the society, and they are provided with efforts for getting betterment in society (Taghipour et al., 2022).

The objective of this study was to identify the effect of CSR, MPs, and OB on SE in the Islamic banking sector of South Punjab. Indeed, there have been different studies on the role of SE in the banking sector, but less attention was paid by the conducted studies to address the issues in the way to promote SE in the Islamic banking sector of South Punjab. In this regard, the theoretical framework of this study was designed to provide practical ways for the improvement of SE in the Islamic banking sector. Significantly, the scope of this study is to improve the SE in the Islamic banking sector of South Punjab.

This study is significant and a hallmark contribution because, to the best of our knowledge, no earlier study was conducted to provide the theoretical and practical implications for the Islamic banking sector of South Punjab related to SE. In this regard, there was a clear gap in the literature, which was addressed in this study. Similarly, this study was designed to provide the theoretical as well as practical implications for the MPs to improve ways to promote SE in the Islamic banking sector of South Punjab. Moreover, the theoretical framework of this study provides a detailed relationship between different variables used for this study to improve SE with OB and CSR.

## Literature review

### Relationship of corporate social responsibility, women empowerment, and social equity

In modern organizations, the role of CSR is important to consider because its function is to take the organization productively (Fiack et al., 2021). However, the management not only is responsible to ensure CSR in the organizational structure but also must be ready to promote SE in society (Islam et al., 2021). On the one hand, some organizations are working effectively to ensure that all the stakeholders are working collectively to provide equal values to the people of the society when they are interacting with the organization (Gryshchenko et al., 2022). However, on the other hand, organizations that failed to establish CSR for SE badly failed to maintain the standard of their working in the target market (Abed et al., 2022). Further, the responsibility of the management is to ensure that all the customers are provided with equal opportunities and equal values when they are in the organization (Fiack et al., 2021). In this regard, organizations that are not effectively working for CSR have failed miserably to remove the barrier of bias from the society, and it has become a critical challenge for the society on a large scale. According to the study of Ahmad et al. (2022), business organizations in advanced and developed countries are working on the policy of CSR to ensure the factor of WE to eliminate gender-based violence in the organization. Moreover, these organizations are working effectively to promote CSR for the organizational performance because it is difficult for the management to deal with the issues of the customer unless the organization is effective (Neal et al., 2022). In this way, the responsibility of the management is increased to ensure the presence of policies of CSR in the organization not only for the betterment of the organization (Fiack et al., 2021) but also to provide equal opportunities and values to the employees and the customers of the organization (Clauß et al., 2022).

H1. There is a relationship between CSR and WE.

H2. There is a relationship between WE and SE.

H3. There is a relationship between CSR and SE.

### Relationship between management practices and social equity

Management practices play an important role in the performance of any organization (Neal et al., 2022). However, in different organizations, the performance of management is different due to different kinds of factors that are affecting the organization (Gryshchenko et al., 2022). It is important to understand that, without effective management, the performance of organizations would be neglected because it is the management that is leading the organization into a product that would be beneficial for all the stakeholders of the organization (Fiack et al., 2021). On the one hand, organizations in developed countries are working to improve the standard of the employees, for which training and workshops are connected to ensure that the performance of all employees reflects the betterment of the organization to provide fair services to the employees (Neal et al., 2022). Moreover, on the other hand, according to de Freitas et al. (2019), some organizations are not working effectively to promote organizational performance. These organizations are badly failing in the target market. Further, there is a critical rule of MPs in SE because SE is fundamental for the stakeholders (Neal et al., 2022); if SE is not provided, it would be difficult for the management to deal with such kinds of problems (Anderson et al., 2018). In this regard, the management is responsible for ensuring that all employees are working in good practices, are well aware of the value of society, and provide better services to each and every member of the society for the collective benefit of the organization. No doubt, organizations with bad management have failed to perform due to a lack of management skills to provide equal opportunities to all their customers (Bariæ, 2017). However, the organizations that are working for the betterment of society are provided with the guidelines to value the customer in a productive way that would be beneficial for them to develop SE (Neal et al., 2022). The organizations in Norway are provided with guidelines to ensure productivity in a way that would benefit customers for social equality and SE (Gryshchenko et al., 2022).

H4. There is a relationship between management practices and SE.

### Relationship between organizational behavior and social equity

**FIGURE 1:**
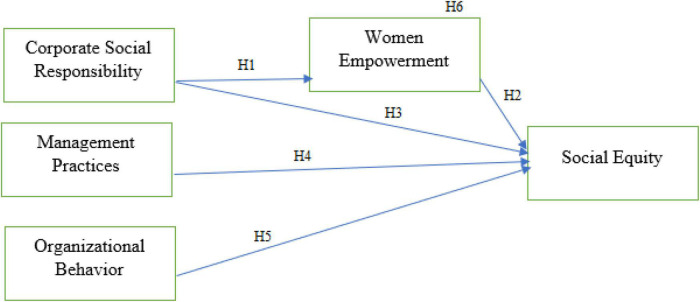
Theoretical framework.

The performance of the organization is when the management is working according to the vision admission of the organization (Gryshchenko et al., 2022). Critically, the performance of different organizations is distinct from each other due to the different kinds of characteristics and traits of the management (Obermayer et al., 2021). In this regard, the responsibility of the management is to ensure that all policies set by the top management are being implemented in the right way for the productivity of the organization according to the vision of the Chief Executive Officer (Gryshchenko et al., 2022). In this way, the organizations that are working with the help of effective management to achieve sustainability in the target market are achieving the goals set by the top management (Bariæ, 2017). However, the responsibility of the management of the organization is to not only generate profit (Fiack et al., 2021) but also ensure that the organization has to work for SE that would be useful for the top management and stakeholders of the organization. In the same manner, the responsibility of the organization is not only to work for SE but also to work to remove all kinds of barriers in the way of equality and to provide effective and sustainable solutions for the benefit of the organization and society as well (Parmentola et al., 2022). Previous studies explained that there is a critical role of management in the organization, and at the same time, these studies also narrated that, if the management is effective and is working with strong OB, then the achievement would be sustainable (Fiack et al., 2021). According to Ivanova-Gongne et al. (2022), the responsibility of the management is to achieve not only sustainability but also sustainability with SE if the OB was improved to the appropriate level. In this way, the performance of the organization not only would be increased (Fiack et al., 2021) but also would be in the right direction to promote the values in society and to work for the society in a better way that would be useful for its productivity (Hida and Dewi, 2021). The theoretical framework of the study is presented in Figure 1.

H5. There is a relationship between OB *and* SE.

H6. There is a mediating role of WE between the relationship of CSR and SE.

## Methodology

### Prepare questionnaire

For this study, the questionnaire was prepared in two different sections. The first section was to collect the demographic information of the respondents. The second section was based on the constructs that were taken from previous studies to measure the relationship presented in the theoretical framework of the study. The questionnaire was based on the five-point Likert scale because it is the best and most appropriate method for getting a response to quantitative data. In this regard, Smart PLS 3 software was selected to measure the response of the respondents to the questionnaire. First, the scale items for CSR were taken from the study of Taghipour et al. (2022). Second, the scale items for MPs were taken from the study of Dathe et al. (2022). Third, the scale items for OB were taken from the study of Neal et al. (2022). Fourth, the scale items for WE were taken from the study of Fiack et al. (2021). Lastly, the scale items for SE were taken from the study of Fiack et al. (2021).

### Data collection process

For this study, data were collected from the population of the customers of the Islamic banking sector of South Punjab. In this regard, the questionnaire was mailed to the respondents in a paid return envelope with a random sampling method because it is the appropriate method to collect data from a large audience. They were asked to provide appropriate and impersonal responses to the questionnaire to contribute to the worth of the study. After the collection of data, 355 questionnaires were considered appropriate out of 700 for data analysis. The respondents were provided with reliable feedback when they provided the questionnaire back to the researcher.

## Findings

### Convergent validity

**FIGURE 2:**
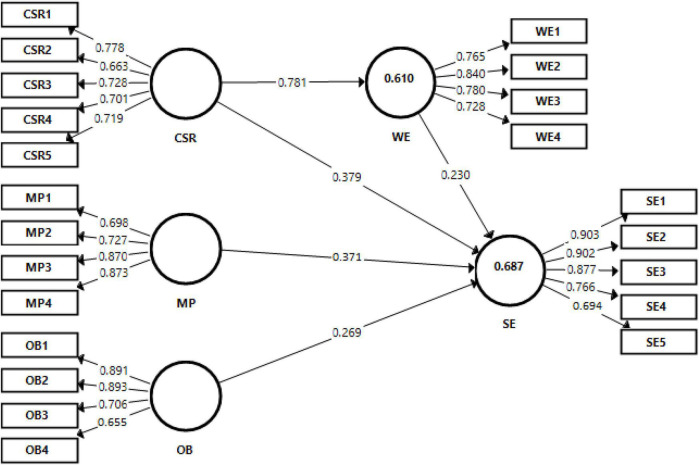
The measurement model.

This section of the study has information related to the convergent validity that is important to check to understand the validity and reliability of the construct (as shown in Figure 2). According to Table 1, all the values of factor loadings were greater than 0.60, which is recommended by Hair et al. (2012) for modern studies. Furthermore, the value of composite reliability for each variable was greater than 0.70, which is also recommended for modern studies. At the same time, the values of average variance extracted were greater than 0.50, which is recommended for modern studies.

**TABLE 1:** Factor loadings, composite reliability (CR), and average variance extracted.

Variables	Items	Loadings	Alpha	CR	AVE
CSR	CSR1	0.778	0.766	0.842	0.517
	CSR2	0.663			
	CSR3	0.728			
	CSR4	0.701			
	CSR5	0.719			
Management practices	MP1	0.698	0.810	0.873	0.634
	MP2	0.727			
	MP3	0.870			
	MP4	0.873			
Organizational behavior	OB1	0.891	0.763	0.852	0.598
	OB2	0.893			
	OB3	0.706			
	OB4	0.655			
Social equity	SE1	0.903	0.850	0.898	0.646
	SE2	0.902			
	SE3	0.877			
	SE4	0.766			
	SE5	0.694			
Women empowerment	WE1	0.765	0.787	0.861	0.607
	WE2	0.840			
	WE3	0.780			
	WE4	0.728			

### Discriminant validity

This section of the study highlights the discriminant validity. It was checked to understand the distinction between variables and constructs taken for this study. In this regard, according to Table 2, all the values of discriminant validity were less than 0.90, which is recommended by Hair et al. (2013) for modern studies. As result, it was identified that there is a distinction between the scale items for each variable.

**TABLE 2:** Discriminant validity.

	CSR	MP	OB	SE	WE
CSR					
MP	0.747				
OB	0.743	0.868			
SE	0.665	0.813	0.802		
WE	0.581	0.814	0.707	0.866	

### The PLS-SEMs results

**FIGURE 3:**
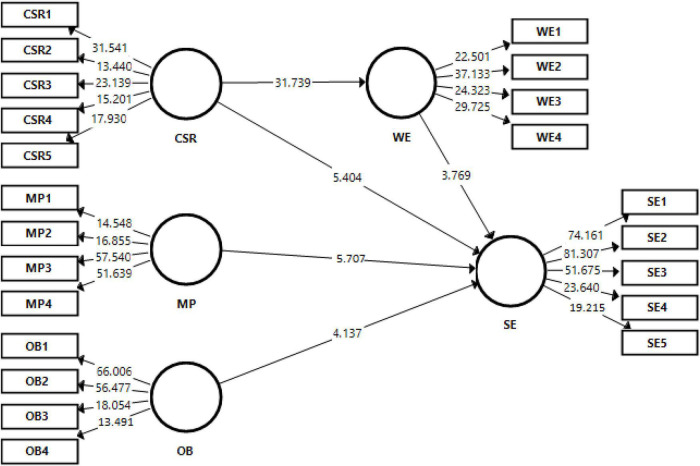
Structural model.

This section of the study has results of direct effects (as shown in Figure 3). H1 was tested for its significance and the results showed that CSR has a significant effect on WE (β = 0.781, *t* = 31.739, and *p* = 0.000); hence, H1 is supported. H2 was tested for its significance, and according to the results, WE has a significant effect on SE (β = 0.230, *t* = 3.769, and *p* = 0.000); hence, H2 is supported. H3 was tested for its significance, and according to the results, CSR has a significant effect on SE (β = 0.379, *t* = 5.404, and *p* = 0.000), and thus, H3 is supported. H4 was tested for its significance, and according to the results, MP has a significant effect on SE (β = 0.371, *t* = 5.707, and *p* = 0.000); hence, H4 is supported. H5 was tested for its significance, and according to the results, OB has a significant effect on SE (β = 0.269, *t* = 4.139, and *p* = 0.000); hence, H5 is supported (as shown in Table 3).

**TABLE 3:** Direct effects.

Hypotheses	Beta	STDEV	*T*-values	*P*-values	Decision
H1. CSR -> WE	0.781	0.025	31.739	0	Supported
H2. WE -> SE	0.230	0.061	3.769	0	Supported
H3. CSR -> SE	0.379	0.070	5.404	0	Supported
H4. MP -> SE	0.371	0.065	5.707	0	Supported
H5. OB -> SE	0.269	0.065	4.137	0	Supported

CSR, corporate social responsibility; WE, women empowerment; SE, social equity; MP, management practices; and OB, organizational behavior.

### Mediation effects

This section of the study has the results of mediation analysis. According to results, WE mediates the relationship between CSR and SE (β = 0.179, *t* = 3.843, and *p* = 0.000); hence, H6 is supported (as shown in Table 4).

**TABLE 4:** Mediation analysis.

Mediation	Beta	STDEV	*T*-value	*P*-value	Decision
H6. CSR -> WE -> SE	0.179	0.047	3.843	0	Supported

## Discussion

According to the results of H1, there is a significant relationship between CSR and WE. According to the results of H2, there is a significant relationship between WE and SE. According to the results of H3, there is a significant relationship between CSR and SE. In this regard, it is critical to understand that CSR has a significant role related to SE. SE is to provide equal value to all members of society related to the banking sector. It is a fact that, with the concept of SE, people are improving their standards of living when they are provided with equal opportunities by the industrial sector. At the same time, SE plays a critical role not only in the industrial sector but in the services sector. Indeed, in developed countries, the services sector is working to provide SE to every stakeholder (Chen et al., 2022). However, in the least developed or third-world countries, such as Pakistan, the role of social activities is important because, in these countries, the values of social activities are not provided according to certain requirements. In this way, more responsibilities of the management of the services sector, particularly the banking sector of Pakistan, involve formulating and developing the policy in any way for the development and enhancement of SE to the advanced level. It is critical to understand that, without the concept of SE, the customers of the banking sector board would go for the alternative if they are not provided with equal opportunities and values from a single banking sector (Obermayer et al., 2021). The top management is responsible for formulating and implementing the strategy in this regard, which must be effective and provide a fruitful outcome in the long term (Kharlanov et al., 2022). Significantly, such opportunities must be considered effectively. Similarly, according to the results of H4 and H5, MPs and OB play a significant role in SE. It is a fact that the organizations that are working in an effective way to improve the standard of living of the people by providing fair services and quality work are fulfilling the requirements of SE. According to the results of H6, there is a significant mediating role of WE in the relationship between CSR and SE. It is a fact that, when the organization is working on the guidelines of WE, then it would be effective for the organization to grow productively by addressing the issues of women (Astrachan et al., 2020; Dathe et al., 2022). Additionally, in this way, the greater the concentration of the organization to improve SE to the advanced level, the more beneficial it is for the community on a large scale.

## Conclusion

This study concludes that there is a critical role of CSR and MPs in developing SE for the women of South Punjab. The study identifies that WE plays an important role as a mediator because, with the help of WE, an advanced level of SE can be achieved by the organization for the women of South Punjab. Importantly, this study demonstrates that, by adopting the guidelines of CSR and by following the role of effective management, the Islamic banking sector of South Punjab can provide more facilities to women to assist them in achieving sustainable development to improve the standards of living with the help of CSR. Therefore, the concentration of the banking sector should improve the MPs and OB for the further improvement of SE for the people. In this way, the community would develop productively with the help of the Islamic banking sector.

## Implications and future directions

This study provides theoretical implications that are important to consider because the gap in the literature is addressed in this study. As, this study highlights the crucial impact of CSR, MPs, and OB in implementation of SE in the Islamic banking sector of South Punjab. In this regard, this study provides guidelines to the management and the administration of the Islamic banking sector of South Punjab to significantly address the issue of SE because it would help the organization to grow in a productive way that would be beneficial for all the stakeholders of the organization in the long run. In a similar manner, this study would lead the Islamic banking sector to the next level in the target market where the customers would be provided with equal opportunities.

This study also provides significant practical implications that are important to consider related to the issues related to SE in the Islamic banking sector of South Punjab. In this regard, it is important to understand that the management of the banking sector should work on workshops and other seminars to create awareness related to SE among employees to ensure that all employees work to reduce this barrier in society. Furthermore, this study also highlights that the role of management should be to anticipate the problems of SE and that there should be a collective effort to reduce these problems in society. Importantly, no earlier study targeted the Islamic banking sector of South Punjab related to the MPs for SE. In this manner, this study provides significant implications to be adopted and implemented by the concerned banking sector.

This study was designed to check the effect of CSR, MPs, and OB to understand the SE status in the Islamic banking sector of South Punjab. Indeed, there are other alternatives in this regard that are important to consider in relation to SE. In this regard, future studies must focus on the role of government policy, employee involvement, and service performance to understand SE in the context of the Islamic banking sector of South Punjab.

## Data availability statement

The original contributions presented in this study are included in the article/supplementary material, further inquiries can be directed to the corresponding authors.

## Ethics statement

Ethical review and approval was not required for the study on human participants in accordance with the local legislation and institutional requirements. Written informed consent for participation was not required for this study in accordance with the national legislation and the institutional requirements.

## Author contributions

All authors listed have made a substantial, direct, and intellectual contribution to the work, and approved it for publication.
